# Oxytocin and Self-Consciousness

**DOI:** 10.3389/fnhum.2016.00067

**Published:** 2016-02-29

**Authors:** Valentina Colonnello, Markus Heinrichs

**Affiliations:** ^1^Laboratory for Biological and Personality Psychology, Department of Psychology, University of FreiburgFreiburg, Germany; ^2^Freiburg Brain Imaging Center, University Medical Center, University of FreiburgFreiburg, Germany

**Keywords:** oxytocin, neuropeptide, consciousness, self, self-other perception

In recent years, the study of self-consciousness from neuroscience and psychobiological perspectives has flourished rapidly. The understanding of the brain areas involved in self-consciousness and the complex intertwining of self-consciousness levels has expanded greatly, and the awareness of neurotransmitter influence on self-consciousness has begun to emerge. Despite this, the neuropeptidergic influences on self-consciousness have been largely ignored However, recent studies of the neuropeptide oxytocin have yielded promising results that further our understanding of the psychobiological foundations of self-consciousness. Here, we argue that the oxytocinergic system plays a role in modulating several levels of self-consciousness, a conclusion that is usually underscored in interpretations of previous studies.

Several studies indicate, that the neuropeptide oxytocin plays a central role in social cognition (Heinrichs and Domes, 2008; Heinrichs et al., 2009). For example, oxytocin administration sharpens mind-reading skills (Domes et al., 2007), enhances understanding of other's perspective (Ditzen et al., 2009), and modulates evaluation of other's trustworthiness (Kosfeld et al., 2005). The cognition of self and other are closely interrelated and partly share the same brain network. For example, the medial prefrontal cortex is involved in other's mental state and personality inference tasks (Mitchell et al., 2002, 2004) as well as in evaluation of one's own personality traits (Johnson et al., 2002). Of note, oxytocin administration modulates the medial prefrontal cortex activity (Sripada et al., 2013).

The understanding of others' perspectives and empathic responses also relies on the ability to recognize self-other differences. Oxytocin administration mediates the activation of brain areas, e.g., the posterior temporal sulcus (Pincus et al., 2010; Gordon et al., 2013), that are involved in mentalizing and processing self-other distinction (Decety and Lamm, 2007). Oxytocin is probably involved in both other- and self-related processing.

Two fundamental issues regarding the oxytocinergic system and self-consciousness are of specific relevance. First, oxytocin is synthesized in the paraventricular and supraoptic nuclei of the hypothalamus and processed to the posterior pituitary lobe, through axonal projections, for release into peripheral circulation. In addition, dendrites release oxytocin into extracellular space, from where it diffuses to several distant areas in the brain. Oxytocin receptors have been found in several brain areas, including the periacqueductal gray, putamen, insula, amygdala, nucleus accumbens, and prefrontal cortex (Sofroniew, 1983; Tribollet et al., 1992; Meyer-Lindenberg et al., 2011). Of note, oxytocin diffuses from evolutionarily ancient areas primarily involved in arousal states and emotion expression to more recent distal brain areas involved in cognitively mediated processes.

The second relevant issue concerns the definition of self-consciousness. There is still no consensus on what self-consciousness is or how it works. Over the past century, scholars from different disciplines and theoretical approaches have proposed several models of consciousness (Panksepp, 1998; Damasio, 1999; Gallagher, 2000; Metzinger, 2003; Morin, 2006; Edelman et al., 2011; Vandekerckhove and Panksepp, 2011), which for the sake of brevity are not discussed here.

Here, we focus on the most recent, prominent psychobiological and evolutionarily-based perspectives, that conceptualize consciousness levels as developing in a continuum throughout phylogenesis (Tulving, 1985; Vandekerckhove and Panksepp, 2009, 2011). In this view, three interdependent levels of self-consciousness are identifiable: (i) the non-reflective, affectively rich anoetic (unknowing) consciousness, which is believed to involve the basal subcortical system, extending from brainstem to hypothalamus to central thalamic nuclei; (ii) noetic (knowing) learning-based consciousness, which relies on activating the lower subcortical ganglia and upper limbic cortical midline structures; and (iii) reflective, metacognitive autonoetic (self-knowing) consciousness, which represents a recent achievement of human evolution and relies on associative neocortical brain areas.

Experiential anoetic consciousness occurs without the cognitively mediated self-observation of one's own action. It is conceptualized as direct, unreflective affective and sensorial-perceptual experience of the world and self; it represents a primary form of first-person phenomenal “self-experience” and is rooted in the subcortical emotional systems that humans share with lower animals (Vandekerckhove and Panksepp, 2009). Although one's attention is apparently toward the external environment rather than toward oneself, this basic level of consciousness still implies the presence of a minimal unifying implicit instance of self that enables the individual to perceive and to approach, or avoid, the surrounding environment, based on affective experience (Tulving, 1985; Vogeley and Fink, 2003; Vandekerckhove and Panksepp, 2009).

Cross-species studies suggest, that oxytocin influences anoetic consciousness. Specifically, oxytocin administration is associated with changes in affective experience and modulation of unreflective/instinctual proximity of the self toward unfamiliar others. For example, oxytocin modulates social affiliation (Nelson and Panksepp, 1998), facilitates approach toward unfamiliar pups in female rats (Pedersen et al., 1992), enhances social motivation in adult male rats and mice (Lukas et al., 2011), reduces separation distress vocalizations in abruptly isolated rat pups (Insel and Winslow, 1991), enhances confidence behavior in chicks (Panksepp, 2009), while facilitating partner-preference formation in voles (Insel and Hulihan, 1995). Given the limited self-reflective abilities in animals, these findings stress the role of oxytocin in influencing the unreflective anoetic form of consciousness and related instinctual emotional behaviors.

In humans, oxytocin administration facilitates the sensitivity and processing of explicit and masked emotional expressions (Leknes et al., 2012; Domes et al., 2013; Prehn et al., 2013; Kanat et al., 2015) and words with evolutionarily self-relevant values (Heinrichs et al., 2004; Unkelbach et al., 2008), prior to their full cognitive evaluation. Presumably, oxytocin facilitates the processing of evolutionarily self-relevant social stimuli and regulates the approach to novel, self-unrelated social stimuli by increasing the salience of social stimuli (Prehn et al., 2013) while reducing stress-reactivity (Heinrichs et al., 2003) and promoting feelings of self-confidence (Colonnello and Heinrichs, 2014).

Oxytocin administration likely affects noetic levels of self-consciousness. This level of consciousness is rooted in lower anoetic levels and is associated with learning-based self-awareness and the recognition of oneself as “me,” that is, the object of one's own observation and the perception of “me” as distinct from “other.” We found, that oxytocin influences implicit self-description: oxytocin administration facilitated the tendency to associate self-related words with positive adjectives in healthy men during an implicit association task (Colonnello and Heinrichs, 2014). In addition, intranasal oxytocin administration sharpens explicit self- and other-recognition during the presentation of videos showing photos of one's own face morphing into those of an unfamiliar individual, and vice versa. Specifically, participants who received intranasal administration of oxytocin were facilitated in the self-other distinction of one's own and other's facial features, regardless of the morphing direction. This effect was associated with rating one's own face and other's faces as comparably pleasant, indicating the facilitation of acceptance of self and others (Colonnello et al., 2013).

Oxytocin's role in enhancing distinctions between self- and other-related stimuli is corroborated by a study on pain perception. In this study, participants who received oxytocin exhibited different responses to visualizations of self- and other-directed painful stimulation. They rated their imaginings of personally experienced pain as less stressful than their thoughts of pain experienced by others (Abu-Akel et al., 2015), which suggests a link between oxytocin-induced dampening of stress reactivity, enhanced self-other distinction, and increased attention to others' needs.

The most complex level of self-consciousness, referred to as autonoetic comprises metacognitive tasks, reflection on oneself and one's experience, and mental time traveling in one's own past and future (Suddendorf and Corballis, 1997; Vandekerckhove and Panksepp, 2009). Empirical studies have indicated, that the oxytocin system modulates autonoetic self-consciousness. As Cardoso and colleagues demonstrated, increased levels of oxytocin facilitate the recollection of personal memories and positive social affiliation memories (Cardoso et al., 2014), as well as the tendency to rate oneself with positive personality traits in self-report questionnaires (Cardoso et al., 2012). Notably, oxytocin's influences on autonoetic levels of consciousness are based on individual differences and attachment styles (Bartz et al., 2011). Future studies should investigate whether the effects of oxytocin on autobiographical memories and self-processing might also be linked to an increase in sensitivity toward one's own attachment representations, regardless of their valence.

While the current sparse data converge in indicating that oxytocin is among the neuromodulators implicated in self-consciousness, it is worth stressing that this research area is still in its infancy and further studies are warranted. But what specific research paradigms would deepen the understanding of oxytocin's role in self-consciousness? Which clinical conditions presenting changes in self-consciousness would benefit from integrating treatments with oxytocin administration?

Undoubtedly, the neuropeptide oxytocin is drawing clinicians' attention due to its effects on several aspects in the social domain. As we previously proposed (Colonnello et al., 2013; Colonnello and Heinrichs, 2014), studies of oxytocin's effects should consider changes not only in one's perceptions of others but also in the processing of self-related information. From the evolutionary and neuroscience perspective, it is plausible that oxytocin affects self-relatedness processing thanks to involvement of the subcortical-cortical midline structures, (Northoff and Panksepp, 2008). Further, at a higher cognitive level, oxytocin likely influences self- and other-related cognition by modulating the brain network activity involved in the shared neural representations of others and self (Lombardo et al., 2010).

Thus, oxytocin's influences on several aspects of self-consciousness and on the interdependence between self-consciousness levels in clinical conditions characterized by alteration of self and other processing cry out for further research attention. The current paucity of data on the oxytocin's effects on noetic self-consciousness also encourages further investigation of its influences on self-other distinction.

Of note, self-recognition and the sense of self-ownership are among the mechanisms implicated in functional empathic responses. Thus, such investigations would be crucial to furthering our understanding of oxytocin's role in the relationship between self-other distinction and the ability to shift from self-oriented to other-person-oriented perspectives, as well as its role in self-other distinction, and prosocial behavior, especially in light of the close link between interoception and empathy (Ernst et al., 2013). In addition, it would be important to address questions regarding oxytocin's effects on the noetic knowledge of self-agency (i.e., perception of being the author of one's own actions) that is likely to be altered in various clinical conditions (Belayachi and Van der Linden, 2010; Synofzik et al., 2010). The potential effects of oxytocin administration should also be studied in individuals with dysmorphic disorders and in patients with neuropsychological disorders displaying altered “noetic” explicit knowledge of their own body boundaries.

Given that levels of self-consciousness unfold over development and that ontogenetic development recapitulates the phylogenetic development, elucidating oxytocin's influences on the interdependence between self-consciousness levels would also be crucial. Because oxytocin plays a role in social bond formation and in regulating stress responses (Heinrichs et al., 2003) and its influences on autonoetic levels vary as a function of prior attachment experiences (Bartz et al., 2011), the relation between early social environment, alterations in central oxytocin transmission, and development of noetic and autonoetic self-consciousness warrants extensive investigation through longitudinal studies.

Finally, future studies should devote effort to addressing the potential therapeutic role of the oxytocin system (Heinrichs and Domes, 2008; Heinrichs et al., 2009; Meyer-Lindenberg et al., 2011). By buffering self-disclosure and “re-editing” of self-with-others experiences in psychotherapy, oxytocin administration may help create an affective psychobiological context for facilitating therapeutic changes.

## Author contributions

All authors listed, have made substantial, direct and intellectual contribution to the work, and approved it for publication.

### Conflict of interest statement

The authors declare that the research was conducted in the absence of any commercial or financial relationships that could be construed as a potential conflict of interest.
